# Investigating the Effects of Diet-Induced Pre-Diabetes on the Functioning of Calcium-Regulating Organs in Male Sprague Dawley Rats: Effects on Selected Markers

**DOI:** 10.3389/fendo.2022.914189

**Published:** 2022-07-11

**Authors:** Karishma Naidoo, Phikelelani S. Ngubane, Andile Khathi

**Affiliations:** School of laboratory Medicine and Medical Sciences, Discipline of Human Physiology, University of KwaZulu-Natal, KwaZulu- Natal, South Africa

**Keywords:** pre-diabetes, calcium-regulating organs, high-fat high carbohydrate diet, homeostasis, normocalcaemia

## Abstract

Derangements to the functioning of calcium-regulating organs have been associated with type 2 diabetes mellitus (T2DM), a condition preceded by pre-diabetes. Type 2 diabetes has shown to promote renal calcium wastage, intestinal calcium malabsorption and increased bone resorption. However, the changes to the functioning of calcium-regulating organs in pre-diabetes are not known. Subsequently, the effects of diet-induced pre-diabetes on the functioning of calcium-regulating organs in a rat model for pre-diabetes was investigated in this study. Male Sprague Dawley rats were separated into two groups (n=6, each group): non-pre-diabetic (NPD) group and a diet-induced pre-diabetic (DIPD) group for 20 weeks. After the experimental period, postprandial glucose and HOMA-IR were analysed in addition to plasma and urinary calcium concentrations. Gene expressions of intestinal vitamin D (VDR), intestinal calbindin-D9k, renal 1-alpha hydroxylase and renal transient receptor potential vanilloid 5 (TRPV5) expressions in addition to plasma osteocalcin and urinary deoxypyridinoline concentrations were analysed at week 20. The results demonstrated significantly increased concentrations of postprandial glucose, HOMA-IR and urinary calcium in addition to unchanged plasma calcium levels in the DIPD group by comparison to NPD. Renal TRPV5, renal 1-alpha hydroxylase, intestinal VDR and intestinal calbindin-D9k expressions were increased in the DIPD group by comparison to NPD. Furthermore, plasma osteocalcin levels were increased and urine deoxypyridinoline levels were decreased in the DIPD group by comparison to NPD. These observations may suggest that calcium-regulating organs compensate for the changes to calcium homeostasis by inducing increased renal calcium reabsorption, increased intestinal calcium absorption and decreased bone resorption followed by increased bone formation.

## Introduction

Urban lifestyle and the chronic consumption of diets which contain high fat and carbohydrate content has shown to promote the development of type 2 diabetes mellitus (T2DM), a condition which is preceded by pre-diabetes (1). Pre-diabetes is an intermediate state of hyperglycaemia with glycaemic parameters above the homeostatic range yet below the threshold for diagnosis of clinical diabetes (1). Pre-diabetes is associated with the simultaneous presence of insulin resistance and β-cell dysfunction (2). In 2017, the International Diabetes Federation (IDF) reported that 352 million people worldwide were diagnosed with pre-diabetes, while it is further estimated that by 2045 the prevalence of pre-diabetes is expected to increase by 8.3% (2). While T2DM is often associated with macro- and microvascular complications, studies have shown that T2DM impairs calcium homeostasis by disrupting the functioning of calcium-regulating organs, namely the intestine, kidney and bone (3, 4).

Fluxes of calcium between the small intestine, bone and kidney are controlled by parathyroid hormone (PTH), calcitonin and calcitriol (5). Calcium-regulating organs participate in supplying calcium to the blood and removing it from blood when necessary (4). The small intestine is the site where dietary calcium is absorbed, the bone serves as a calcium reservoir and the kidneys regulate urinary calcium excretion (6). Several studies have shown physiological changes to calcium-regulating organs in T2DM individuals (4, 7, 8). Type 2 diabetes mellitus promotes impaired intestinal calcium absorption, renal calcium wasting and bone deterioration (4). Furthermore, it also leads to dysregulation of calciotropic hormones, thereby worsening the already impaired functioning of calcium-regulating organs (6). The maintenance of calcium homeostasis is important because calcium modulates many important functions (4). Calcium is responsible for bone mineralization, hormone communication, regulation of the nervous system and muscle tone (5). By interrupting calcium homeostasis, processes within the body that are dependent on calcium would be impaired as well as conditions such as hypocalcaemia and osteoporosis may develop (4).

Previous studies in our laboratory developed a high fat high carbohydrate (HFHC) diet-induced pre-diabetic animal model which mimics the human condition of pre-diabetes (9, 10). Several studies using this model have revealed that various complications seen in T2DM begin in the pre-diabetic state (9, 11). While the changes to the functioning of calcium-regulating organs have been well documented in the diabetic state, these changes have not yet been investigated during the pre-diabetic state (12, 13). Hence, the aim of this study is to determine the effects of diet-induced pre-diabetes on the functioning of calcium-regulating organs in male Sprague Dawley rats.

## Materials and Methods

### Animals

Sprague-Dawley male rats (150-180 g) were bred and housed at the University of KwaZulu-Natal’s Biomedical Research Unit (BRU). The protocol for animal experimentation and conditions were followed according to the Animal Research Ethics Committee of the University of KwaZulu-Natal (ETHICS#: AREC/00003627/2021). Procedures involving animals care were conducted in conformity with the institutional guidelines for animal care of the University of KwaZulu-Natal. The animals were maintained under standard conditions of a constant room temperature (22± 2°C), carbon dioxide content (<5000 p.m.), relative humidity (55± 5°C) and illumination (12 h light/dark cycle, with lights on at 7am). The noise levels in the room were maintained below 65 decibels and the animals had access to rat chow and water *ad libitum*. An experimental acclimatization interval of one week was carried out, whereby the rats were fed standard rat chow (Meadows Feeds, South Africa) and tap water, prior to the induction of pre-diabetes (10).

### Outcome and Outcome Measures

The primary outcome was evaluation of intestinal calcium transport, renal calcium transport and bone turnover. The secondary outcome was evaluation of body calcium status, glucose tolerance and insulin resistance. Intestinal calcium transport was measured by evaluating intestinal VDR and intestinal calbindinD9k expression. Renal calcium transport was measured by evaluating renal TRPV5 and renal 1-alpha hydroxylase expression. Bone turnover was measured by evaluating plasma osteocalcin and urine deoxypyridinoline levels. Body calcium status was measured by evaluating plasma calcium and urine calcium levels. Glucose tolerance and insulin resistance was measured by evaluating OGTT, plasma insulin and HOMA-IR.

### Induction of Pre-Diabetes

The rats were randomly assigned following simple randomisation procedures (computerised random numbers) to 1 of 2 two groups (n=6, per group) and fed their respective diets for an experimental period of 20 weeks. Six rats per group were the minimum amount of animals needed to achieve statistical significance according to the resource method equation (14). Experimental pre-diabetes was induced in the animals using a previously described protocol by 10 (10). Basically, one group was fed a standard rat diet and tap water, whereas the other group was fed a HFHC diet and 15% fructose supplemented water (AVI Products (Pty) Ltd, Waterfall, South Africa), for the purpose of inducing pre-diabetes. After 20 weeks, the animals were tested to confirm pre-diabetes using the criteria from the American Diabetes Association. Animals with a fasting blood glucose (FBG) concentration of 5.6 to 7.1 mmol/L, oral glucose tolerance test (OGTT) 2-h glucose concentration of 7.1–11.1 mmol/L and plasma triglycerides concentration greater than 2 mmol/L were regarded as pre-diabetic. The animals that were fed the standard diet were also tested at week 20 to confirm normoglycaemia.

### Experimental Design

This study comprised of two groups, namely a diet-induced pre-diabetic (DIPD) group and non-pre-diabetic (NPD) group (n=6, in each group). The animals that consumed the standard rat chow for 20 weeks and did not have pre-diabetes were regarded as the NPD group, whereas the animals that consumed the HFHC diet for the same number of weeks and diagnosed with pre-diabetes, were regarded as the DIPD group. Baseline OGTT and HOMA-IR could not be performed due to analysis being limited to changes in glycaemic parameters at week 20 after dietary intervention, in addition blood samples were not obtained at baseline.

### Oral Glucose Tolerance Response

At week 20, an OGTT was conducted following glucose loading, to determine the glucose tolerance response of animals subjected to the chronic ingestion of the HFHC diet. The OGT responses were monitored in the animals according to a well-established protocol (9, 10, 15). Briefly, after a 12 hour fast, glucose levels were measured (time, 0 min) in all animals. Thereafter, the animals were loaded with glucose (glucose; 0.86 g/kg, p.o) through an oral gavage (18-gauge gavage needle, 38mm long curved with 21/4 mm ball end). To measure glucose concentration, blood was collected using the tail-prick method (16). Glucose concentrations were measured by a OneTouch select glucometer (Lifescan, Mosta, Malta, United Kingdom). The glucose concentrations were measured at 15, 30, 60, and 120 minutes following glucose loading.

### Urine Collection, Blood Collection and Tissue Harvesting

At the end of the experimental period, all animals were housed individually in Makrolon polycarbonate metabolic cages (Techniplats, Labotec, South Africa) for a 24-hour urine collection period. After the 24-hours, urine samples from all animals were collected and stored in a Bio Ultra freezer (Labotec, Umhlanga, South Africa) at -80°C, thereafter the animals were anaesthetized with Isofor (100 mg/kg) (Safeline Pharmaceuticals (Pty) Ltd, Roodeport, South Africa) for 3 minutes *via* a gas anaesthetic chamber (Biomedical Resource Unit, UKZN, South Africa). While the rats are unconscious, blood was collected by cardiac puncture and then injected into individual pre-cooled heparinized containers. The blood was centrifuged (Eppendorf centrifuge 5403, LGBW Germany) at 4°C, 503 g for 15 minutes. Plasma was separated from blood and stored at -70°C in a Bio Ultra freezer (Labotec, Umhlanga, South Africa) until biochemical analysis as previously described by 10. Following blood collection, the kidney and small intestine were removed and placed in pre-cooled Eppendorf containers and snap-frozen in liquid nitrogen before storage in a Bio Ultra freezer (Snijers Scientific, Tilburg, Netherlands) at − 80°C. Of note, plasma, urine, kidney and intestinal tissue were obtained from a previous study which had ethical approval.

### HOMA-IR Index

The homeostatic model assessment (HOMA) was used to measure insulin resistance from fasting blood glucose and insulin levels (17). The HOMA-IR index was calculated using the HOMA2 Calculator v2.2.3 program (18.). Values < 1.0 = insulin sensitive, >1.9 = early insulin resistance, > 2.9= significant insulin resistance.

### Biochemical Analysis

Plasma calcium, urinary calcium and creatinine concentrations were measured with an autoanalyzer (IDEXX VetLab station, Hoofddorp, Netherlands). The plasma insulin, plasma osteocalcin and urinary deoxypyridinoline concentrations were measured using separate specific ELISA kits according to the manufacturer’s instructions (Elabscience and Biotechnology, Wuhan, China).

### Quantitative Real-Time PCR

The harvested kidney and small intestine tissue was subjected to RNA extraction using a ReliaPrep tissue Miniprep system (Promega, USA). The purity and concentration of RNA was determined by Nanodrop 2000 (Thermo Scientific, Roche, South Africa). A purity ratio (A260/A280) of 1.7-2.1 was considered acceptable for conversion to cDNA. Synthesis of cDNAs was performed by reverse transcription reactions with 2 μg of total RNA using GoTaq^®^ 2-Step RT-qPCR System as a cDNA synthesis kit (Promega, USA) as described by the manufacturer.

The ROCHE light cycler SYBR Green I master mix was used for amplication according to the manufacturer’s instructions on the ROCHE light cycler system. The primer sequences (Metabion, Germany) used in this study can be found in **Table 1** below. The cycling conditions were: Pre-incubation was carried out at 95°C for 60s, followed by a 3-step amplication of 45 cycles at 95°C for 15s, 60°C for 30s, and 72°C for 30s. Melting was effectuated at 95°C for 10s, 65°C for 60s and 97°C for 1s. Furthermore, cooling was achieved at 37°C for 30s. Glyceraldehyde-phosphate dehydrogenase (GAPDH) was used as the housekeeping gene. Gene expression values were represented using the 2^-ΔΔCt^ relative quantication method.

**Table 1:** List of primers used in this study.

Gene of interest	Sequence
**TRPV5**	Forward: 5’-TGTGAGCCATTTGTAGGTCAG-3’ Reverse: 5’-GAGGTTGTGGGAACTTCGA-3’
**CYP27B1(1-alpha hydroxylase)**	Forward: 5’-CACCCATTTGCATCTCTTCC -3’ Reverse: 5’-GATGGATGCTCCTCTCAGGT -3’
**VDR**	Forward: 5′-GTGACTTTGACCGGAACGTG-3′ Reverse: 5’- ATCATCTCCCTCTTACGCTG -3’
**S100G(CalbindinD9k)**	Forward: 5’CCCGAAGAAATGAAGAGCATTTT-3’ Reverse: 5’-TTCTCCATCACCGTTCTTATCCA-3’
**GAPDH**	Forward: 5’-AGTGCCAGCCTCGTCTCATA-3’ Reverse: 5’-GATGGTGATGGGTTTCCCGT-3’

**Table 1** List of primers used in this study

### Statistical Analysis

All data was expressed as mean ± S.E.M. Statistical comparisons were performed with Graph Pad InStat Software (version 5.00, Graph Pad Software, Inc., San Diego, California, USA) using the student t test. A value of p < 0.05 was considered statistically significant.

## Results

### Oral Glucose Tolerance Test

**Figure 1:**
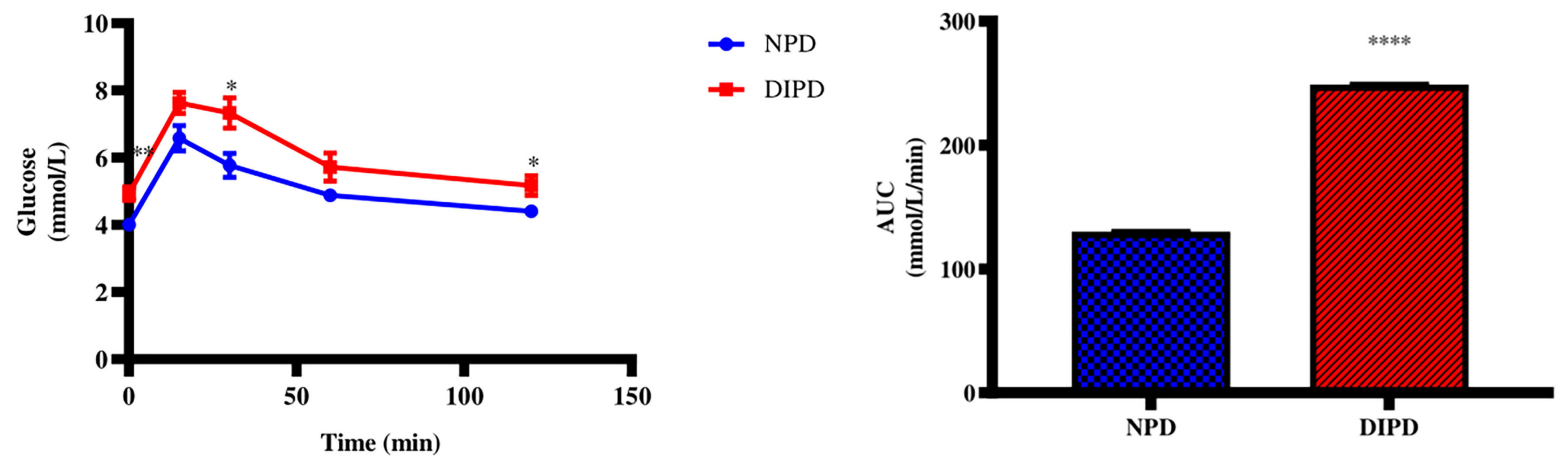
The OGT response and AUC values in the non-pre-diabetic (NPD) group and diet-induced pre-diabetic group (DIPD) (n=6, per group). Values are presented as mean ± SEM. *=p < 0.05, **=p < 0.01, ****= p < 0.0001 by comparison with NPD.

The OGTT and Area under curve (AUC) were analysed in the non-pre-diabetic (NPD) group and diet-induced pre-diabetic (DIPD) group after the experimental period (n=6, per group). The results (**Figure 1**) showed that at time 0, the FBG concentration significantly (p= 0.0020) increased in the DIPD group by comparison to the NPD group. At 120 min post-load of glucose, the glucose concentrations of the DIPD group was significantly (p= 0.0386) increased by comparison to the NPD group. Furthermore, the AUC (**Figure 1**) was significantly (p=<0.0001) higher in the DIPD group by comparison to NPD.

### Homeostatic Model Assessment for Insulin Resistance

The HOMA-IR values were calculated from the fasting plasma glucose and insulin concentrations after the experimental period (n=6, per group). The results (**Table 2**) showed that the fasting plasma glucose (p=< 0.0001) and insulin (p=< 0.0001) concentrations were significantly higher in the DIPD group by comparison to the NPD group. The HOMA-IR value for NPD was within the insulin-sensitive range (< 1.0) while the DIPD group had a significantly (p=< 0.0001) higher HOMA-IR value compared to the NPD which was in the range of significant insulin resistance (>2.9).

**Table 2:** Plasma glucose, plasma insulin concentrations and HOMA-IR indices in the non-pre-diabetic (NPD) group and diet-induced pre-diabetic group (DIPD) (n=6, per group).

Groups(n=6)	Plasma glucose (mmol/L)	Plasma insulin(ng/mL)	HOMA-IR values
NPD	4.40 ± 0.20	3.47 ± 0.12	0.68 ± 0.05
DIPD	6.72 ± 0.12****	10.87 ± 0.06****	3.24 ± 0.06****

Values are presented as mean ± SEM. ****= p < 0.0001 in comparison with NPD.

**Table 2** Plasma glucose, plasma insulin concentrations and HOMA-IR indices in the non-pre-diabetic (NPD) group and diet-induced pre-diabetic group (DIPD) (n=6, per group). Values are presented as mean ± SEM. ****= p<0.0001 in comparison with NPD

### Plasma Calcium and Urinary Calcium From 24-Hour Urine Samples

**Figure 2:**
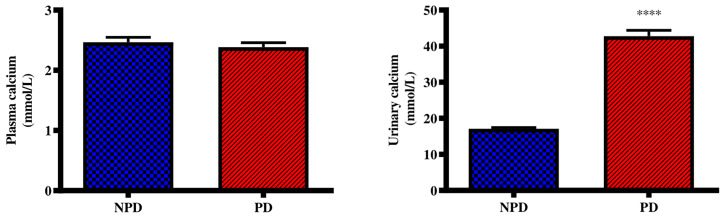
Plasma and urinary calcium concentrations in the non-pre-diabetic (NPD) group and diet-induced pre-diabetic group (DIPD) (n=6, per group). Values are presented as mean ± SEM. ****= p < 0.0001 by comparison with NPD.

Plasma and urinary calcium concentrations were analysed in the non-pre-diabetic (NPD) group and diet-induced pre-diabetic (DIPD) group after the experimental period (n=6, per group). The results (**Figure 2**) showed that there was no significant (p=<0.0001) change to plasma calcium concentration in the DIPD group by comparison to NPD. The urinary calcium concentration (**Figure 2**) were significantly (p= <0.0001) higher in the DIPD group by comparison to NPD.

### Evaluation of Bone Turnover Through Plasma Osteocalcin and Urine Deoxypyridinoline Levels

**Figure 3:**
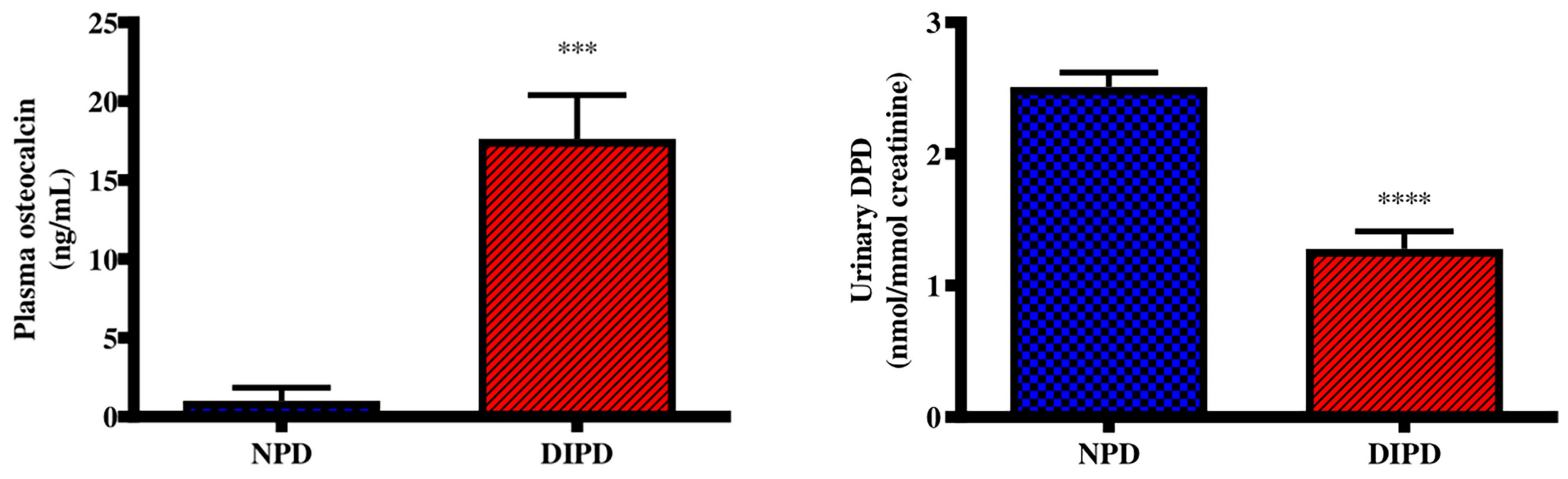
Evaluation of bone turnover through plasma osteocalcin and urine deoxypyridinoline concentrations in the non-pre-diabetic (NPD) group and diet-induced pre-diabetic (DIPD) group (n=6, per group).Values are presented as mean ± SEM. ***=p < 0.001, ****=p < 0.0001 by comparison with NPD.

Plasma osteocalcin and urine deoxypyridinoline concentrations were analysed in the non-pre-diabetic (NPD) group and diet-induced pre-diabetic (DIPD) group after the experimental period (n=6, per group). The results (**Figure 3**) showed that plasma osteocalcin concentration was significantly higher (p= 0.0002) in the DIPD group by comparison to NPD. The urinary deoxypyridinoline concentration (**Figure 3**) was significantly (p=<0.0001) lower in the DIPD group by comparison to the NPD group. A Pearson’s correlation analysis was performed in both non-pre-diabetic (NPD) and diet-induced pre-diabetic (DIPD) rats between plasma osteocalcin and HOMA-IR. The results (**Supplementary Table 1**) showed that plasma osteocalcin levels were positively correlated (r=0.87, p=0.02) with HOMA-IR in the pre-diabetic state.

### Evaluation of Renal Calcium Transport Through Renal TRPV5 and 1-Alpha Hydroxylase Expression

**Figure 4:**
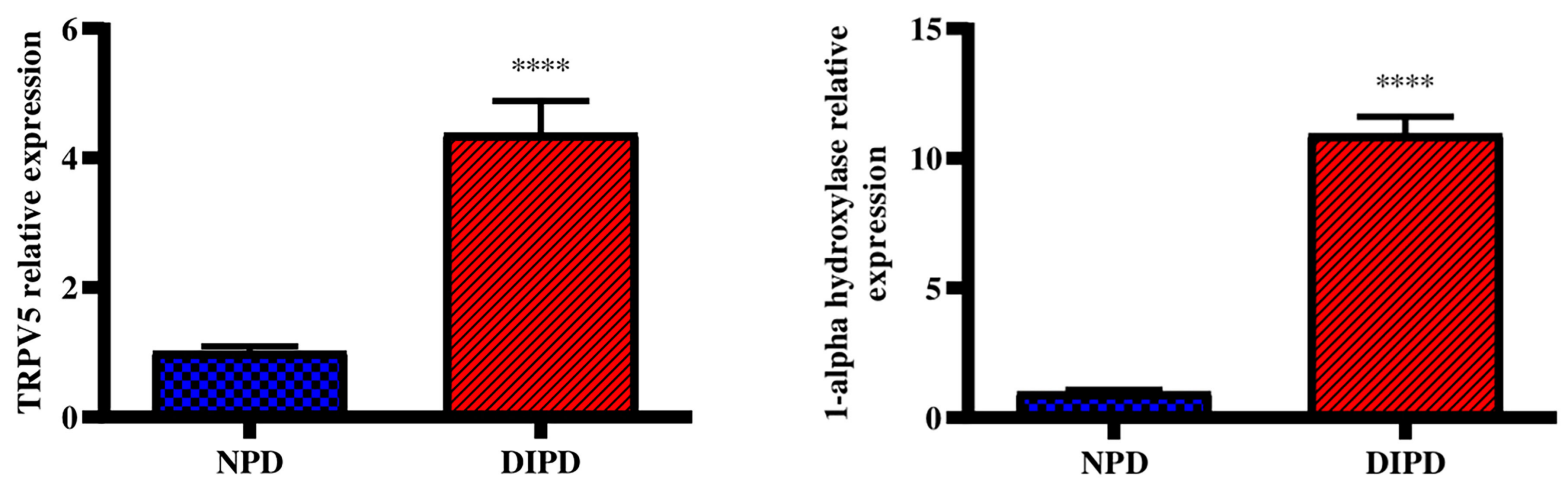
Evaluation of renal calcium transport through renal TRPV5 and 1-alpha hydroxylase gene expression in the non-pre-diabetic (NPD) group and diet-induced pre-diabetic (DIPD) group (n=6, per group).Values are presented as mean ± SEM. ****= p < 0.0001 by comparison to NPD.

Renal Transient receptor potential cation channel subfamily V5 (TRPV5) gene expression was analysed in the non-pre-diabetic (NPD) group and diet-induced pre-diabetic (DIPD) group after the experimental period (n=6, per group). The results (**Figure 4**) showed that the relative expression of renal TRPV5 was significantly (p=<0.0001) increased by 3.89-fold in the DIPD group relative to the NPD group. The relative expression of renal 1-alpha hydroxylase (**Figure 4**) was significantly (p=<0.0001) increased by 10.96-fold in the DIPD group relative to the NPD group.

### Evaluation of Intestinal Calcium Transport Through Intestinal VDR and CalbindinD9k Expression

**Figure 5:**
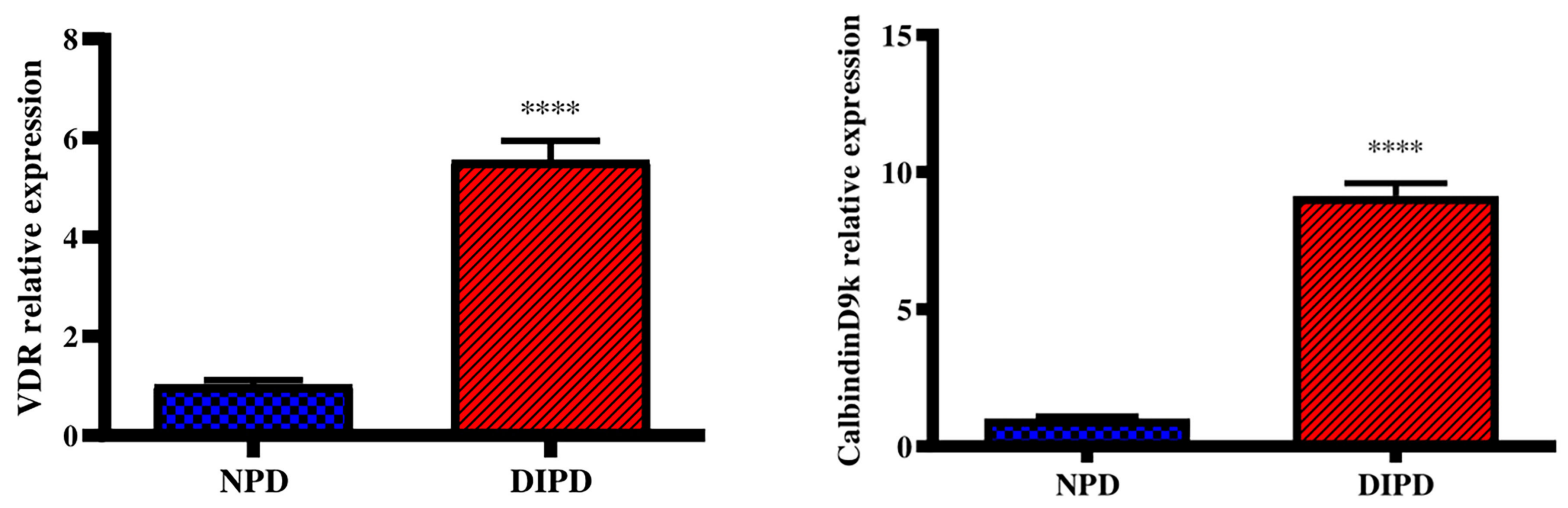
Evaluation of intestinal calcium transport through intestinal VDR and calbindinD9k gene expression in the non-pre-diabetic (NPD) group and diet-induced pre-diabetic (DIPD) group (n=6, per group).Values are presented as mean ± SEM. ****= p < 0.0001 by comparison to NPD.

Intestinal vitamin D receptor (VDR) gene expression was analysed in the non-pre-diabetic (NPD) group and diet-induced pre-diabetic (DIPD) group after the experimental period (n=6, per group). The results (**Figure 5**) showed that the relative expression of intestinal VDR was significantly (p=<0.0001) increased by 5.55-fold in the DIPD group relative to the NPD group. The relative expression of intestinal calbindinD9k expression (**Figure 5**) was significantly (p=<0.0001) increased by 9.13-fold in the DIPD group relative to the NPD group.

## Discussion

Several studies have shown that the functioning of calcium-regulating organs are disturbed in T2DM (4, 19). However, no studies have been conducted to assess the functioning of calcium-regulating organs during the pre-diabetic state. Hence, this study aimed to investigate the effects of diet-induced pre-diabetes on the functioning of calcium-regulating organs, namely the kidney, intestine and bone. The current study found a significant change in the functioning of calcium-regulating organs induced by the pre-diabetic state. In this study, there was increased concentrations of postprandial glucose, plasma insulin and HOMA-IR index in the DIPD group by comparison to NPD. Furthermore, we have found that the pre-diabetic state induced by HFHC diet increases the levels of urinary calcium as well as the expressions of renal TRPV5, renal 1-alpha hydroxylase, intestinal VDR and intestinal calbindinD9k. The pre-diabetic group have also presented increased plasma osteocalcin, decreased urinary deoxypyridinoline concentrations, but unchanged plasma calcium. The findings highlight the physiological compensatory role of calcium-regulating organ systems in the pathogenesis of pre-diabetes.

Pre-diabetes is characterised as a combination of impaired glucose tolerance (IGT) and impaired fasting glucose (IFG) which can be attributed to moderate insulin resistance in insulin-dependent tissues (16). Blood glucose levels must be constantly maintained within a physiological range including a fasting glucose level of less than 5.6 mmol/L and postprandial glucose level of less than 7.8 mmol/L (20). In the postprandial state of normal glucose tolerant (NGT) individuals, blood glucose concentration increases and insulin is secreted to enhance glycogenesis and inhibit glycogenolysis (1). As a result, plasma glucose levels are maintained followed by plasma insulin levels returning towards the homeostatic range (21). However, in the pre-diabetic state endogenous glucose production is excessive before eating and fails to appropriately suppress after eating in pre-diabetic individuals (22). This is due to impaired insulin-induced peripheral glucose uptake in insulin-dependent tissue (10). This accounts for fasting plasma glucose, insulin, postprandial glucose levels and HOMA-IR been higher in pre-diabetic individuals by comparison to NGT individuals (23). In this study, there was a significant increase in the postprandial glucose concentration at 120 min, AUC and HOMA-IR value in DIPD group by comparison to NPD. The results corroborated with previous findings that have shown significantly higher plasma glucose, insulin, 2-hour postprandial glucose levels and HOMA-IR in pre-diabetic patients by comparison to NPD (2, 24). In the DIPD group, the elevated plasma insulin, impaired fasting glucose and HOMA-IR value in the range of insulin resistance may suggest that there is some insulin resistance from peripheral tissue against the uptake of glucose. High dietary fat promotes an increase in circulating triacylglyceride which breakdown to free fatty acids (FFA) (25). The increase in FFAs around insulin-dependent tissue results in insulin resistance which decreases glucose uptake resulting in compensatory hyperinsulinemia, as seen in the DIPD group (10). Elevated plasma glucose concentrations and the onset of insulin resistance in T2DM has shown to interfere with the functioning of calcium-regulating organs in the diabetic state.

Calcium plays a crucial role in various physiological processes and plasma calcium levels are kept within a narrow range through the interplay of calcium-regulating organs (26). Calcium-regulating organs maintain plasma calcium levels by regulating renal calcium reabsorption, bone turnover and intestinal calcium absorption (6). Previous studies have shown decreased plasma calcium concentrations in type 2 diabetic patients by comparison to normoglycaemic individuals (19, 27). These observations suggested that renal dysfunction and abnormal vitamin D metabolism were responsible for inducing a state of hypocalcaemia in the diabetic state (26). However, other studies have shown normal plasma calcium levels in diabetic individuals (28, 29). These studies have stated that calcium-regulating organs compensate for the reduced plasma calcium levels by inducing an increase in intestinal calcium absorption, renal calcium reabsorption and bone resorption (30). In this study, the DIPD group had no significant change to plasma calcium levels by comparison to the NPD group. The findings in this study coincided with prior literature that have shown no significant change to plasma calcium levels by comparison to diabetic patients (31, 32). The possible reason for no significant change to plasma calcium levels in the pre-diabetic state may have been due to calcium-regulating organs compensating for the changes to plasma calcium levels. Interestingly, it may be speculated that during the pre-diabetic state there may be counter-regulatory mechanisms remaining that are aimed at the maintenance of calcium homeostasis. The early insulin resistance and intermediate hyperglycaemia in pre-diabetes may activate these counter-regulatory mechanisms, where compensation may be the initial response to disturbances to calcium homeostasis. However, it may be observed that serum alterations of calcium seem to be observed only after renal injury progression culminating in diabetes mellitus.

The kidneys contribute to calcium homeostasis by adjusting the reabsorption and excretion of filtered calcium (6). Disturbances in renal calcium reabsorption can lead to excessive urinary calcium excretion and kidney stone formation (8). TRPV5 is a calcium channel which mediates calcium reabsorption in the kidney and plays an important role in the regulation of urinary calcium (33). Studies have reported elevated urinary calcium levels along with decreased renal TRPV5 expression in diabetes (34, 35). Studies have also shown that decreased renal TRPV5 expression was associated with reduced renal calcium reabsorption (36, 37). These observations suggested that hyperglycaemia-induced renal damage may have downregulated renal TRPV5 expression (37). The downregulation in renal TRPV5 expression promotes renal calcium wastage and hypocalcaemia in diabetics (38). In addition, elevated urinary calcium levels have shown to result from intestinal hyperabsorption of calcium and excessive bone resorption in T2DM (39). In this study, the DIPD group had significantly increased urinary calcium concentrations in the range of hypercalciuria by comparison to NPD. This was accompanied by a significant increase in the expression of renal TRPV5 in the DIPD group by comparison to NPD. This study’s results corroborated with previous studies that have shown elevated urine calcium and an upregulation in renal TRPV5 expression in T2DM patients (40, 41). The increased urine calcium may have occurred as a result of kidney damage in the pre-diabetic state, which may have decreased the ability of the kidneys to reabsorb calcium. There may have been other contributors to the increased urine calcium such as increased intestinal calcium absorption and bone resorption (28). Therefore, the kidneys may try to compensate for the increased plasma calcium by excreting it into urine. However, the simultaneous increase in urinary calcium excretion and renal TRPV5 may suggest a compensatory mechanism against renal calcium wastage. The increased renal TRPV5 expression in the DIPD group may have promoted increased renal calcium reabsorption from urinary filtrate. Interestingly, renal TRPV5 expression is regulated by vitamin D, which is known to be catalyzed to its active form in the kidney (5).

Renal-1 alpha hydroxylase is mainly expressed in the proximal convoluted tubules and is the key enzyme involved in the synthesis of calcitriol (42). Disturbances in kidney function and vitamin D metabolism can lead to excessive urinary calcium excretion and hyperparathyroidism (26). Studies have shown that a loss of kidney function in T2DM leads to a decline in circulating plasma calcitriol concentrations (43, 44). Renal injury and the accumulation of metabolites in the diabetic kidney contribute to 1-alpha hydroxylase inhibition and lower circulating calcitriol levels (43). In this study, the DIPD group had significantly increased renal-1 alpha hydroxylase expression by comparison to the NPD group. The findings of this study corroborated with previous studies that have shown an upregulation in renal 1-alpha hydroxylase expression in diabetic patients (42, 45). However, the findings of this study contrasted other studies that have shown decreased renal-1 alpha hydroxylase expression in the diabetic state (43, 44). The upregulation in renal-1 alpha hydroxylase in the DIPD group may suggest that there is an increased demand to synthesis calcitriol in the pre-diabetic state. The kidneys may compensate for the hypercalciuria by upregulating the expression of renal-1 alpha hydroxylase, in order to maintain normal plasma calcium levels. In addition, the regulation of renal-1 alpha hydroxylase is dependent on the calciotropic hormones (46). It is evident that renal cells still appear be responsive to calciotropic hormones in the pre-diabetic state, in attempt to conserve plasma calcium levels.

Intestinal calcium absorption is a crucial physiological process for maintaining calcium homeostasis (30). The small intestine is the site where dietary calcium is absorbed and can physiologically adapt according to the conditions of the body (47, 48). Efficient absorption of calcium in the small intestine is dependent on the expression of calcium-binding proteins and vitamin D receptor (VDR) (47, 49). Vitamin D metabolites regulate calcium absorption in the intestine through activation of the vitamin D receptor (VDR) which results in increased expression of calcium transport proteins including calbindinD9k (47). Type 2 diabetes is associated with profound deterioration of calcium metabolism, partly from impaired intestinal calcium absorption (6, 50). Previous studies in diabetic rats reported that the reduction in intestinal calcium absorption occurred concurrently with decreases in VDR and calcium-binding protein calbindin-D9k in the enterocytes (4, 51). It was noted that intestinal VDR and calcium-binding proteins were downregulated due to impaired production of calciotropic hormones in T2DM (6). Subsequently, the ability of the intestine to adapt to disturbances to low plasma calcium levels is compromised during the diabetic state (6). However, other studies have shown an upregulation in intestinal calcium transporter expression in diabetic rats (47, 52). The increased intestinal VDR number promoted increased VDR-calcitriol complexes and increased intestinal calcium transport (6). Hence, the present study investigated intestinal VDR and calbindinD9k expression to evaluate intestinal calcium transport. In this study, there was a significant increase in intestinal VDR and calbindinD9k expression in the DIPD group by comparison to the NPD group. The findings of this study contrasted previous results that have shown a downregulation in intestinal VDR and calbindinD9k expression in the diabetic state. The elevated intestinal VDR and calbindinD9k expression in the DIPD group may suggest that there is an increase in intestinal calcium absorption. The upregulation of calcium transport genes in the intestine of the DIPD group may have been a compensatory response to renal calcium wastage.

Bone regulates plasma calcium levels by releasing calcium through a process known as bone resorption and storing calcium through a process known as bone formation (53). Bone formation is coupled to bone resorption, where increased bone resorption is followed by increased bone formation (5). An imbalance between bone resorption and bone formation may result in bone diseases including osteoporosis (54, 55). Bone resorption and formation can be determined indirectly by measurement of plasma concentrations of bone markers (29). These markers include bone matrix components released into circulation during bone formation or resorption (56). Osteocalcin is a marker of bone formation, whereas deoxypyridinoline is a marker of bone resorption (56). Some studies have shown increased bone turnover in type 2 diabetic patients, where bone resorption exceeds formation (20, 57). This was evidenced by decreased plasma osteocalcin levels and increased deoxypyridinoline levels (4, 58). These observations suggested that during the diabetic state there is an increased demand to mobilize calcium from bone to compensate for hypocalcaemia; however the normal bone coupling process becomes compromised (4, 58). Hyperglycaemia has shown to decrease bone formation by inhibiting osteoblast synthesis and differentiation (28). However, other studies have reported increased bone formation in type 2 diabetes (20, 59). It was stated that hyperinsulinemia shifts the balance between bone formation and resorption in favour of bone formation (56). Hence, the present study focused on investigating the levels of plasma osteocalcin and urine deoxypyridinoline in the pre-diabetic state, to evaluate bone turnover. In this study, the DIPD group had increased plasma osteocalcin concentration and decreased urinary deoxypyridinoline concentration in the DIPD group by comparison to NPD. The findings of this study corroborated with previous results that have shown increased plasma osteocalcin levels and decreased urinary deoxypyridinoline levels (59, 60). These observations may suggest that there is increased bone formation and decreased resorption in the pre-diabetic state. The increased bone formation and decreased bone resorption may have been induced by calciotropic hormones to compensate for hypercalcaemia. Interestingly, studies have shown that increased intestinal calcium absorption by calciotropic hormones in the diabetic state may overcompensate for renal calcium wastage inducing a state of hypercalcaemia (61, 62). In this study the intestine may have overcompensated for renal calcium wastage inducing a state of hypercalcaemia. This leads to the speculation that during the pre-diabetic state there may be some resistance in the detection of plasma calcium levels. Subsequently, bone may have suppressed bone resorption and promoted bone formation to compensate for hypercalcaemia. Insulin is an anabolic hormone which has shown to promote bone formation and inhibit bone resorption (59). In the pre-diabetic state, early insulin resistance leads to a compensatory increase in insulin secretion (63). The elevated plasma insulin levels in the DIPD group may have promoted increased bone formation and suppressed bone resorption. Furthermore, previous studies have shown a positive correlation between HOMA-IR and plasma osteocalcin level in diabetic patients (64, 65). It has been demonstrated that osteocalcin can stimulate insulin secretion, acting directly on proliferation and secretion of pancreatic beta-cells (20). Interestingly, there was a positive correlation between plasma osteocalcin and HOMA-IR in the DIPD group. It may be speculated that the elevated plasma osteocalcin concentration in the DIPD group may have been a compensatory response to cope with the early insulin resistance. This may be an early adaptation mechanism for insulin resistance, which fails with the onset of overt T2DM.

The findings elucidated in this study may have the potential to provide an understanding into the physiological processes that occur in calcium-regulating organs during pre-diabetes. From a clinical perspective, pre-diabetes is asymptomatic and many people progress towards the development of T2DM due to being unaware. The findings of this study will not only add to academic knowledge but may serve as a novel marker in the identification of pre-diabetes. This study targets some of the complications and disrupted processes involved in T2DM. Furthermore, these findings may provide an early insight into the pathogenesis involved in the associated complications of T2DM. A future prospective would be to use these findings as insights to understand the possible changes that may occur to pre-diabetic humans.

It is evident that during the pre-diabetic state there are changes to the functioning of calcium-regulating organs which compensate for disturbances to plasma calcium levels. This was made evident by increased urinary calcium levels along with increased expressions of renal TRPV5, renal 1-alpha hydroxylase, intestinal VDR and intestinal calbindinD9k. In addition, there was increased plasma osteocalcin and decreased urinary deoxypyridinoline concentrations along with unchanged plasma calcium in the pre-diabetic state. The normocalcaemia present in the pre-diabetic state may have been conserved due to increased renal calcium reabsorption, increased renal vitamin D activation, increased intestinal calcium absorption and increased bone formation followed by decreased bone resorption.

## Conclusion

Taken together, calcium-regulating organs compensate for renal calcium wastage and are aimed at maintaining normocalcaemia in HFHC diet-induced pre-diabetes. The effects associated with pre-diabetes on calcium-regulating organs are directed towards promoting increased renal calcium reabsorption, increased renal vitamin D activation, increased intestinal calcium absorption and decreased bone resorption followed by increased bone formation. This was evidenced by increased expression of renal calcium transport markers and intestinal calcium transport markers in addition to increased osteocalcin and decreased deoxypyridinoline levels. Collectively, these observations may suggest that calcium-regulating organs compensate for the changes to calcium homeostasis in the pre-diabetic state.

## Limitations

A limitation of the study was the lack of blinding during the diet intervention stage and the lack of baseline measurement for hepatic and muscle insulin resistance marker (OGTT and HOMA-IR). A second limitation to this study is that it only focuses on mRNA expression and not proteins.

## Future Recommendations

In future, a further insight into the mechanisms in which bone turnover by-products participate in glucose homeostasis in the pre-diabetic state should be investigated.

## Data Availability Statement

The original contributions presented in the study are included in the article/**Supplementary Material**. Further inquiries can be directed to the corresponding author.

## Ethics Statement

This animal study was reviewed and approved by the Animal Research Ethics Committee (AREC) of the University of KwaZulu-Natal, Durban, South Africa (AREC/00003627/2021).

## Author Contributions

KN contributed to the study design, conducted the experiments, collected, analysed and interpreted data as well as being involved in writing the manuscript. PN and AK was involved in the conceptualization of the study, study design and editing of the manuscript as well as provide funding. All authors have read the manuscript and approved its submission.

## Funding

This study was funded by the UKZN College of Health Science and the National Research Foundation under the grant number (UID130598).

## Conflict of Interest

The co-author AK of this manuscript is the editor of the Frontiers Journal Thieme (Pre-diabetes and endocrine function)

The remaining authors declare that the research was conducted in the absence of any commercial or financial relationships that could be construed as a potential conflict of interest.

## Publisher’s Note

All claims expressed in this article are solely those of the authors and do not necessarily represent those of their affiliated organizations, or those of the publisher, the editors and the reviewers. Any product that may be evaluated in this article, or claim that may be made by its manufacturer, is not guaranteed or endorsed by the publisher.
