# Supplementary material for: Investigating the Effects of Diet-Induced Pre-Diabetes on the Functioning of Calcium-Regulating Organs in Male Sprague Dawley Rats: Effects on Selected Markers
Source: Front Endocrinol (Lausanne). 2022 Jul 11;13:914189. doi: 10.3389/fendo.2022.914189 (PMC9309376; doi:10.3389/fendo.2022.914189)
Supplement: Supplementary file 1 [file Table_1.pdf]

Osteocalcin-HOMA-IR correlation

Supplementary Table 1 Pearson's correlation between plasma osteocalcin and HOMA-IR in the non-pre-diabetic (NPD) group and diet-induced pre-diabetic group (DIPD) (n=6, per group). Values are depicted as mean ± SEM. *= p<0.05

**Metabolic Parameter**		**Plasma osteocalcin**
**HOMA-IR values**	**NPD**	r= 0.80<br>p= 0.06
	**DIPD**	r= 0.87<br>p= 0.02*
